# From an emerging pathogen to therapeutic challenges: advances in *Achromobacter* species, with a focus on *A. xylosoxidans* and cystic fibrosis

**DOI:** 10.3389/fmicb.2026.1954013

**Published:** 2026-09-24

**Authors:** Yunfeng Xiang, Daoxue Xiong, Xiangjun Liu

**Affiliations:** Department of Pediatrics, Chongqing University Three Gorges Hospital, Wanzhou, Chongqing, China

**Keywords:** *Achromobacter*, *Achromobacter xylosoxidans*, antimicrobial resistance, phage therapy, whole-genome sequencing

## Abstract

*Achromobacter* spp. are aerobic, non-fermentative, Gram-negative opportunistic pathogens belonging to the family Alcaligenaceae, order *Burkholderiales*, and are widely distributed in natural environments such as soil and water. In patients with cystic fibrosis (CF), *Achromobacter* spp. have emerged as increasingly prevalent opportunistic pathogens, with detection rates in respiratory samples rising from approximately 2–3% in the 1990s and early 2000s to 7–10% by the early 2020s in European and North American registries, although reported rates vary considerably by center, age group, and identification method. These bacteria also affect immunocompromised individuals outside the CF population. However, precise diagnosis and treatment of *Achromobacter* infections remain particularly challenging because of progressive difficulties: inaccurate species-level identification, limited clinical breakpoints for most species, and synergistic multidrug resistance mechanisms. This review synthesizes recent epidemiological data, outbreak investigations, and tentative epidemiological cutoff values (ECOFFs) for *A. xylosoxidans* established by EUCAST. We also present an integrated framework that links motility loss, biofilm formation, and resistance development to describe within-host adaptive evolution in the CF airway, and we highlight the role of biofilm-mediated tolerance and persister cells in chronic infection. By synthesizing multidimensional evidence, this review aims to provide a comprehensive reference framework for basic research and clinical management of *Achromobacter*, and to identify priority research directions, including the establishment of standardized identification procedures and the advancement of standardized clinical development of phage therapy.

## 1 Introduction

*Achromobacter* spp. are Gram-negative, strictly aerobic, non-spore-forming, rod-shaped bacteria. They are motile by peritrichous flagella. Taxonomically, the genus belongs to the phylum Pseudomonadota, class Betaproteobacteria, order *Burkholderiales*, and family Alcaligenaceae (Bhowmik and Stubbendieck, 2025; Isler et al., 2020). *Achromobacter* spp. are widely distributed in natural environments such as soil, water, and plant rhizospheres, and are also found in hospital settings (Amoureux et al., 2013). With the widespread use of polyphasic taxonomic methods, the number of formally named species in this genus has rapidly expanded from only a few initially to 22 validly published species (Isler et al., 2020; Meier-Kolthoff et al., 2022).

In recent years, *Achromobacter* spp. have gained increasing clinical attention as emerging opportunistic pathogens. Infections caused by these bacteria are often associated with multidrug-resistant phenotypes, which can limit treatment options (Bhowmik and Stubbendieck, 2025). *Achromobacter xylosoxidans* is the earliest confirmed human pathogen within this genus. The type strain of this species was first isolated from human ear discharge in 1971 (Yabuuchi and Oyama, 1971). For a long time, this genus received relatively limited attention in clinical microbiology, mainly because of low infection rates and difficulties in laboratory identification. However, over the past two decades, advances in molecular identification techniques have helped reveal the clinical importance of this genus. In patients with cystic fibrosis (CF), the detection rate in respiratory samples has risen from about 2–3% to 7–10% (Bhowmik and Stubbendieck, 2025). In non-CF populations, *Achromobacter* spp. are important causes of bacteremia, pneumonia, and catheter-related infections in immunocompromised individuals, and they have also been found in a small number of people without clear immunodeficiency (Bayless et al., 2025).

Despite the growing clinical importance of *Achromobacter*, several key knowledge gaps remain. First, commercial MALDI-TOF MS databases have limited discriminatory power at the species level, making accurate identification difficult in clinical laboratories (Garrigos et al., 2021; Papalia et al., 2020). Second, the Clinical and Laboratory Standards Institute (CLSI) does not provide species-specific clinical breakpoints for *Achromobacter*, and interpretation of susceptibility results therefore relies on breakpoints for “other non-Enterobacterales” (Ray et al., 2025). The European Committee on Antimicrobial Susceptibility Testing (EUCAST) has established specific clinical breakpoints for *A. xylosoxidans* for piperacillin-tazobactam, meropenem, and trimethoprim-sulfamethoxazole, but breakpoints for other *Achromobacter* species remain unavailable (Isler et al., 2020; Ray et al., 2025). Third, the spectrum of virulence factors and the molecular mechanisms of pathogenicity in *Achromobacter* are not fully understood (Acharya et al., 2026). Fourth, large-scale prospective clinical evidence on the optimal treatment of multidrug-resistant strains is lacking (Yabuuchi and Yano, 1981). These gaps represent the core scientific questions in the field and define the main focus of this review.

This review synthesizes recent advances in the taxonomy, identification, clinical epidemiology, resistance mechanisms, virulence factors, and emerging therapeutic strategies of *Achromobacter*. We identify directions for future research and provide a reference framework for the clinical management of *Achromobacter* infections, with particular attention to CF care. We note that the prevalence estimates cited in this section derive primarily from European and North American CF registries, and should be interpreted with caution, as detection rates vary considerably by country, age group, identification method, and CF center; they do not represent a universal temporal trend. We also note that most mechanistic and clinical evidence currently concerns *A. xylosoxidans* and CF patients, and this limitation is reflected in the scope of our analysis.

## 2 Taxonomy, identification methods, and literature search strategy

### 2.1 Taxonomic evolution

The taxonomic evolution of the genus *Achromobacter* is a dynamic and ongoing process. The genus was established by Yabuuchi and Oyama in 1971, with *Achromobacter xylosoxidans* as the type species. The genus name was formally recognized under the International Code of Nomenclature of Bacteria in 1981 (Yabuuchi and Oyama, 1971; Yabuuchi and Yano, 1981). Through methods such as DNA–DNA hybridization, 16S rRNA gene sequencing, and multilocus sequence analysis (MLSA) of housekeeping genes, the taxonomic framework of the genus has been continuously revised and expanded. Several studies have reclassified a number of species previously assigned to the genus *Alcaligenes* into *Achromobacter*, which has markedly increased its species diversity (Yabuuchi et al., 1998). In 2013, Vandamme et al. formally described the new species *A. aegrifaciens*, *A. anxifer*, *A. dolens*, and *A. insuavis* based on four clinical genogroups carrying virulence genes (Vandamme et al., 2013). In the same year, four additional species, *A. animicus*, *A. mucicolens*, *A. pulmonis*, and *A. spiritinus*, were identified from human clinical samples (Vandamme et al., 2013).

Over the past decade, deeper exploration of environmental microbial communities and growing attention to opportunistic pathogens in clinical microbiology laboratories have led to the continuous reporting of novel *Achromobacter* species from niches such as soil, water, plant rhizospheres, and from animal and human clinical specimens. These findings have further enriched the species diversity of the genus. According to the latest records in the List of Prokaryotic names with Standing in Nomenclature (LPSN) database (accessed on 10 August 2026), the genus currently includes 22 validly published species: *A. xylosoxidans* (type species), *A. denitrificans*, *A. insolitus*, *A. marplatensis*, *A. piechaudii*, *A. ruhlandii*, *A. spanius*, the clinically relevant species described in 2013 (*A. aegrifaciens*, *A. animicus*, *A. anxifer*, *A. dolens*, *A. insuavis*, *A. mucicolens*, *A. pulmonis*, *A. spiritinus*), and later reported environmental species such as *A. aestuarii*, *A. kerstersii*, and *A. seleniivolatilans* (Meier-Kolthoff et al., 2022; Spilker et al., 2013; Papalia et al., 2020). Additional recently described species include *A. agilis*, *A. aloeverae*, *A. deleyi*, *A. pestifer*, *A. sediminum*, and *A. veterisilvae* (Spilker et al., 2013; Papalia et al., 2020).

Globally, at least 10 species have been reported from human infections such as cystic fibrosis. These include *A. xylosoxidans*, *A. ruhlandii*, *A. denitrificans*, *A. insuavis*, *A. dolens*, *A. marplatensis*, *A. pulmonis*, *A. aegrifaciens*, *A. mucicolens*, and *A. animicus* (Isler et al., 2020; Spilker et al., 2013; Papalia et al., 2020). The rapid growth in species numbers and the increasing frequency of clinical isolation underscore the ecological shift of this genus at the environment-human interface and place higher demands on its accurate identification. Because biochemical phenotypes among members of the genus are highly similar and the 16S rRNA gene is highly conserved, molecular identification results are often inconsistent. This situation highlights the urgent need for a more refined taxonomic and identification framework.

### 2.2 Laboratory identification methods: from traditional to molecular era

Laboratory identification of *Achromobacter* species has long been a technical challenge in clinical microbiology. Traditional biochemical methods and commercial MALDI-TOF MS databases often misidentify different species of this genus as *A. xylosoxidans*. Two main reasons account for this. First, biochemical phenotypes are highly similar among closely related species, making reliable differentiation based on phenotypic methods difficult (Gomila et al., 2014). Second, commercial MALDI-TOF MS databases contain only a limited number of reference spectra for these species, which results in insufficient discrimination of mass spectral peaks (Ray et al., 2025).

The study by Garrigos et al. is particularly informative. The researchers constructed a custom MALDI-TOF MS reference database using 205 isolates from 18 *Achromobacter* species, all confirmed by *nrdA* sequencing. They then validated the database with 167 additional isolates. The custom database achieved an identification accuracy of 99.4% (166/167, score ≥2.0) for the 18 species. In contrast, the commercial Bruker database achieved only 50.9% accuracy (85/167). Moreover, 17.3% of the isolates (29/167) in the commercial database were misidentified as other species even though they had scores ≥2.0 (Garrigos et al., 2021). This finding clearly indicates that the bottleneck in species-level identification by MALDI-TOF MS is not an inherent limitation of the technology platform. Rather, it lies in the coverage of the reference database and the completeness of the mass spectral profiles.

When MALDI-TOF MS identification is inconclusive or when higher resolution is needed, molecular methods serve as indispensable complementary tools. Sequencing of the *nrdA* gene provides higher resolution than 16S rRNA gene sequencing at the species level and has been recommended by several studies as the reference method for *Achromobacter* species identification (Garrigos et al., 2021). Multilocus sequence typing (MLST) and whole-genome sequencing (WGS) offer tools with even higher resolution for population structure analysis, reconstruction of interspecies phylogenetic relationships, and molecular epidemiological investigations (Spilker et al., 2012; Ridderberg et al., 2012). Ray et al. performed whole-genome sequencing on 109 clinical isolates and demonstrated that WGS can simultaneously deliver species identification, antimicrobial resistance gene screening, and virulence factor analysis (Ray et al., 2025). WGS has therefore become the preferred technical platform in research laboratories.

Gabrielaite et al. carried out a longitudinal analysis of 101 *Achromobacter* genomes from 51 CF patients over a 20-year infection period. They identified mutational signatures and patterns of genetic variation linked to within-host adaptive evolution and pinpointed key genetic loci associated with increased antibiotic resistance (Gabrielaite et al., 2021). Another longitudinal genomic surveillance study, based at a Danish CF center, further clarified potential transmission routes among patients and the population dynamics of *Achromobacter* (Gabrielaite et al., 2021). Together, these studies provide genomic-level evidence, from the perspectives of within-patient microevolution and population-level transmission, for understanding the mechanisms of persistent colonization and the epidemiological features of *Achromobacter* in the CF airway.

### 2.3 Literature search strategy and selection criteria

To ensure transparency, we conducted a structured literature search. We searched PubMed, Web of Science, and Scopus from database inception to August 2026. The search terms included “*Achromobacter*,” “*Achromobacter xylosoxidans*,” “*cystic fibrosis*,” “antimicrobial resistance,” “virulence factors,” “phage therapy,” “whole-genome sequencing,” “identification,” and “breakpoints.” We included peer-reviewed original research articles, systematic reviews, and international registry reports. We excluded case reports with fewer than three patients, non-peer-reviewed sources, and studies lacking species-level identification unless they provided relevant clinical or epidemiological context. We prioritized primary studies over narrative reviews. The last search was performed on 15 August 2026. This review represents a critical narrative synthesis that applies systematic search principles to reduce selection bias, rather than a formal systematic review with meta-analysis.

## 3 Clinical significance and epidemiology

### 3.1 Infections in patients with cystic fibrosis

Patients with cystic fibrosis (CF) represent the most important high-risk group for *Achromobacter* infections, and this population has been the focus of the most intensive clinical research on this genus (Bhowmik and Stubbendieck, 2025; Bayless et al., 2025). However, reported detection rates vary widely across studies and centers, reflecting differences in case mix, microbiological methods, and clinical definitions; the figures cited below should not be generalized without local validation. Bhowmik and Stubbendieck provided a comprehensive overview of *Achromobacter* as an emerging CF pathogen. They noted that the detection rate of this bacterium in CF patients has risen markedly over the past two decades, and that the risk of colonization is higher in adults than in children (Bhowmik and Stubbendieck, 2025).

Before proceeding, we define key terms. “Isolation” or “detection” means recovery of the organism from a clinical specimen. “Colonization” means repeated isolation over at least two visits within 12 months without clinical evidence of active infection. “Chronic infection” means persistent isolation for more than 12 months with associated clinical decline or inflammatory markers. “Active infection” means isolation accompanied by acute clinical deterioration, such as pulmonary exacerbation. These definitions align with those used in the European Cystic Fibrosis Society Patient Registry and the cited longitudinal studies (Kerem et al., 2023; Bar-On et al., 2025).

The European multicenter registry study published by Kerem et al. is one of the largest epidemiological analyses to date. This study included 38,795 CF patients, of whom 2,093 reported *Achromobacter* infection, yielding a prevalence of 5.4%. Prevalence varied considerably among countries and increased with age. A particularly noteworthy finding was that lung function and body mass index (BMI) in patients infected with *Achromobacter* were comparable to those in patients infected with *Pseudomonas aeruginosa*, whereas patients co-infected with both pathogens showed more severe lung function decline and worsening nutritional status (Kerem et al., 2023). This “dual-pathogen association” indicates that co-infection with both organisms is linked to worse outcomes. Whether this reflects a true synergistic pathogenic mechanism, baseline disease severity, or treatment-related factors remains unclear, because observational registry data cannot establish causality (Kerem et al., 2023).

A single-center, 14-year longitudinal study by Bar-On et al. further refined the understanding of *Achromobacter* infection. The investigators stratified patients into three groups: those with a single positive culture, those with intermittent infection, and those with chronic infection. The detection rate of *Achromobacter* rose from 0 to 6.1% during the study period. At the time of first detection, the median forced expiratory volume in 1 s (FEV₁) of infected patients was 81% (IQR 46–94), compared with 90% (IQR 72–99) in uninfected patients (*p* < 0.001). Two years after initial detection, the decline in lung function—measured as annual FEV₁ decline (both percentage predicted and absolute volume loss per year)—was markedly greater in the chronic and intermittent infection groups than in the single positive culture group. The frequency of annual acute exacerbations also increased significantly after infection, from a median of 2.5 (range 0–8) to 3.0 (range 0–9) per year (*p* = 0.036) (Bar-On et al., 2025). The dose–response relationship revealed in this study provides stronger causal evidence for the pathogenicity of *Achromobacter* than detection rates alone.

Recio et al. performed a longitudinal study in CF patients and were the first to systematically evaluate the quantitative impact of *Achromobacter* respiratory tract infection on lung function. They found that the annual rate of FEV₁ decline was significantly greater in the infected group than in the control group (−2.3% ± 1.6% vs. -1.1% ± 0.9% per year; *p* = 0.02), and was even more pronounced in the chronically infected subgroup (−2.9% ± 1.9% vs. -1.1% ± 0.9%; *p* = 0.04) (Recio et al., 2018). The mean number of annual pulmonary exacerbations was also higher in the infected group (1.9 ± 0.9 vs. 1.1 ± 0.8; *p* = 0.03) (Recio et al., 2018). However, it is important to note that some earlier, smaller studies did not observe a significant association between chronic colonization and accelerated lung function decline, and differences in virulence among species have not been consistently confirmed (Garrigos et al., 2021). This inter-study inconsistency may arise from species misclassification due to differing identification methods, limited sample sizes, and heterogeneity in the definitions of “infection” and “colonization.” These factors together point to a core need: future multicenter studies should uniformly adopt molecular methods for species-level identification and establish a standardized framework for defining infection and colonization.

### 3.2 Infection Spectrum in non-CF patients

In non-CF populations, *Achromobacter* mainly affects immunocompromised individuals, but immunocompetent persons can also be infected. A large-scale retrospective study by Bayless et al. covering three Mayo Clinic sites over a ten-year period (2013–2023) identified 1,598 clinical isolates of *Achromobacter* and provided the most comprehensive picture of non-CF infections to date. In the bloodstream infection (BSI) subgroup, the mean patient age was 58.3 years (±16.7), the median Charlson comorbidity index was 5 (IQR 3–8), 59.6% of patients were immunocompromised, and the most common underlying condition was hematopoietic stem cell transplantation (23.1%). The most frequent source of BSI was vascular catheter-related infection, accounting for 57.7, and 48.1% of episodes were polymicrobial. This study also confirmed that piperacillin-tazobactam, carbapenems, and trimethoprim-sulfamethoxazole (TMP-SMX) can serve as preferred options for empirical therapy (Bayless et al., 2025). A recent case report of community-acquired *A. piechaudii* bacteremia in a healthy child further illustrates this possibility (Xiang et al., 2026).

Regarding infection types, pneumonia and bacteremia are the most common clinical presentations. Endocarditis, catheter-related bloodstream infection, meningitis, ocular infections, urinary tract infections, and peritonitis have also been reported (Isler et al., 2020). Most infections show features of healthcare-associated infections (HAI), and the use of intravenous catheters is often closely linked to pathogenesis (Bayless et al., 2025). Case reports have documented recurrent *A. xylosoxidans* bacteremia and peripherally inserted central catheter (PICC)-related infections in immunocompromised patients, including critically ill patients with prolonged hospitalization (Isler et al., 2020), which underscores the key role of catheter management in infection control. In addition, infections have been reported in immunocompetent hosts and special groups such as neonates (Turel et al., 2013), suggesting that the host range of this bacterium may be broader than previously recognized.

### 3.3 Infection spectra of clinically relevant species

*A. xylosoxidans* predominates in clinical infections, accounting for 42–90% of isolates across different patient populations (Spilker et al., 2013; Kerem et al., 2023; Garrigos et al., 2022). This species is associated with a wide range of infection sites. In CF patients it mainly colonizes the respiratory tract, while in non-CF patients it frequently causes bloodstream infections (often catheter-related), urinary tract infections, and skin and soft tissue infections, together forming a diverse infection spectrum (Bayless et al., 2025; Ray et al., 2025). Other species are detected at considerably lower frequencies, and their infection site distributions differ. *A. ruhlandii* has been isolated from CF respiratory specimens at rates of 2.8–8.3% (Spilker et al., 2013; Bocharova et al., 2024). *A. insuavis* is mainly detected in the respiratory tract of CF patients (5.5–8%) (Spilker et al., 2013; Garrigos et al., 2022). *A. denitrificans* has an isolation rate of approximately 1.3–6.4% and has occasionally been reported in bloodstream infections and in non-CF populations (Bayless et al., 2025; Ray et al., 2025). *A. dolens* and *A. pulmonis* are occasionally detected in respiratory specimens (Vandamme et al., 2013; Vandamme et al., 2013). Species such as *A. piechaudii*, *A. marplatensis*, and *A. animicus* have been reported at isolation rates of 1.1–2.4% in CF respiratory samples, accounting for only 1–2 isolates in large cohort studies (Papalia et al., 2020), and evidence for their clinical pathogenicity remains limited (Isler et al., 2020). Geographic variation exists in species distribution, and some species that are considered rare may be underestimated because of insufficient identification capacity (Ray et al., 2025; Garrigos et al., 2022).

### 3.4 Epidemiological trends and outbreaks

Taken together, the data presented above highlight several notable trends in the epidemiology of *Achromobacter* infections. Regarding detection rates and pathogen spectrum, the isolation rate of this bacterium in CF patients has continued to rise. In some centers, it has become a major non-fermentative Gram-negative pathogen, with isolation rates second only to *Pseudomonas aeruginosa* and *Stenotrophomonas maltophilia* and now exceeding those of the *Burkholderia cepacia* complex (Bcc) (Bocharova et al., 2024). The risk of colonization increases with age and is markedly higher in adults (≥18 years) than in children (Bhowmik and Stubbendieck, 2025; Bocharova et al., 2024). In a Russian CF cohort, the detection rate of *Achromobacter* spp. was 4.5% among patients aged 9–18 years (Bocharova et al., 2024), while European registry data showed that prevalence peaked at age 20–30 years (Kerem et al., 2023). Geographically, species distribution and detection rates vary considerably across countries and centers (Kerem et al., 2023; Garrigos et al., 2022), likely reflecting differences in sampling strategies, identification methods, and infection control practices.

Notably, the widespread use of CFTR modulator therapy is reshaping these epidemiological patterns. A longitudinal study by Mianowski et al. in 198 French adults with CF showed that after one year of elexacaftor-tezacaftor-ivacaftor (ETI) treatment, colonization rates for several *Achromobacter* species declined significantly (*p* < 0.001) (Mianowski et al., 2024). This suggests that in the era of highly effective CFTR modulator therapy, detection rates may no longer follow a unidirectional upward trend. Instead, they may enter a new phase that is profoundly influenced by therapeutic intervention. However, although ETI reduces bacterial burden, it does not completely clear the pathogen (Milczewska et al., 2024), and the long-term clinical impact of persistent colonization remains to be seen.

In addition, outbreaks in healthcare settings have provided important clues about transmission dynamics. Taken together, outbreak investigations point to three core features of *Achromobacter* transmission: water or solution contamination as the source, contact transmission as the main route (for example, patient contact with contaminated sensors or connection tubing), and invasive procedures (including surgery and double-J stent placement) as key risk factors (Tian et al., 2024; Said et al., 2023). Removal of the contaminated source and enhanced disinfection of devices are central measures for ending such outbreaks.

## 4 Antimicrobial resistance

### 4.1 Intrinsic resistance mechanisms: the dual defense of efflux pumps and β-lactamases

*Achromobacter* spp. exhibit intrinsic resistance to multiple antimicrobial agents, which makes them one of the more challenging pathogens in clinical practice (Isler et al., 2020). Ray et al. performed a comprehensive phenotypic and genotypic analysis of 109 clinical isolates and systematically revealed the resistance mechanisms present in this genus (Ray et al., 2025).

Among the efflux pump systems, AxyXY-OprZ is the most clinically relevant RND (Resistance-Nodulation-Division) type multidrug efflux pump. Ray et al. performed whole-genome sequencing and phenotypic susceptibility testing on 109 clinical isolates. Genotypic analysis identified the RND-type efflux pump genes *axyXY-oprZ* in 80% of isolates, and phenotypic testing confirmed that the presence of this efflux system correlated with resistance to fluoroquinolones, cephalosporins, and aminoglycosides (Ray et al., 2025). Notably, isolates lacking this system (e.g., *A. mucicolens*) were susceptible to all tested aminoglycosides, supporting a causal role for AxyXY-OprZ in intrinsic resistance (Ray et al., 2025). Chalhoub et al. conducted a longitudinal analysis of 41 clinical isolates of *A. xylosoxidans* and *A. insuavis* from CF patients. Using quantitative RT-PCR and fluorescent substrate uptake assays, they showed that efflux activity was significantly enhanced in strains with elevated MICs for amikacin or azithromycin. Notably, expression of AxyXY-OprZ is controlled by the AxyZ transcriptional regulator (Chalhoub et al., 2022). Bador et al. demonstrated that under tobramycin selective pressure, inactivating mutations in AxyZ can lead to overexpression of the efflux pump, shifting the phenotype of clinical isolates from intrinsic to a higher level of acquired resistance (Bador et al., 2017).

Chromosomally encoded β-lactamases constitute a second line of defense. The genotypic analysis by Ray et al. showed marked differences in resistance gene profiles among species: *A. xylosoxidans* carried the highest number of resistance genes, including multiple class D (OXA-type) β-lactamases and the class A β-lactamase *blaAXC*, whereas *A. mucicolens* had the fewest resistance genes and lacked efflux pump genes (Ray et al., 2025). Papalia et al. studied the genomic characteristics of *Achromobacter* genogroup 20 and identified a novel OXA-type β-lactamase, OXA-1238, which differs from the known OXA-114a by 85 amino acids, with only 69% sequence identity. This finding suggests that the diversity of β-lactamases in this genus may be substantially underestimated (Papalia et al., 2025).

Although existing studies have outlined the dual resistance framework of efflux pumps and β-lactamases, several aspects remain to be clarified. These include the complete molecular mechanisms governing efflux pump expression, the substrate hydrolysis profiles of different OXA enzyme variants, and their quantitative association with carbapenem resistance phenotypes (Ray et al., 2025; Bador et al., 2017).

### 4.2 Species-specific resistance differences and the threat of pandrug-resistant strains

Different *Achromobacter* species show marked differences in their resistance profiles and the number of resistance genes they carry, and this has important implications for species-level precision anti-infective therapy. The comprehensive phenotypic and genotypic analysis of 109 clinical isolates by Ray et al. showed that non-xylosoxidans species generally had lower MIC 50 and MIC 90 values for carbapenems and β-lactamase inhibitor combinations than *A. xylosoxidans*[*A. xylosoxidans*: imipenem MIC50/MIC90 2/>8 μg/mL, meropenem 0.5/>8 μg/mL, piperacillin-tazobactam ≤8/64 μg/mL; non-xylosoxidans: imipenem 1/2 μg/mL, meropenem 0.5/4 μg/mL, piperacillin-tazobactam ≤8/≤8 μg/mL] (Ray et al., 2025). This species-dependent resistance feature, from a clinical perspective, confirms the need for accurate species-level identification.

A major threat in the evolution of resistance is the acquisition of carbapenemase genes. Kirikae et al. detected *A. xylosoxidans* strains carrying *blaNDM-1* in Myanmar, showing the potential of *Achromobacter* to achieve high-level carbapenem resistance by acquiring exogenous carbapenemase genes (Kirikae et al., 2025). Multidrug-resistant *Achromobacter* strains have been reported repeatedly in CF patients. Pandrug-resistant strains, that is, isolates resistant to nearly all available classes of antimicrobial agents, have also been documented (Isler et al., 2020) and pose a serious challenge to current treatment strategies.

At present, the global prevalence, transmission mechanisms, and quantitative association with clinical outcomes of acquired carbapenemase genes in *Achromobacter* remain to be systematically clarified. This should be a priority direction for future multicenter genomic surveillance studies (Ray et al., 2025; Kirikae et al., 2025).

### 4.3 Activity of new antimicrobial agents

Given the highly resistant phenotypes of *Achromobacter*, evaluating the activity of new antimicrobial agents has become an urgent need to guide clinical treatment. In their review, Isler et al. grouped new agents with potential anti-*Achromobacter* activity into novel β-lactam/β-lactamase inhibitor combinations (BL/BLI), cefiderocol, novel tetracycline derivatives, and novel aminoglycosides (Isler et al., 2020).

For novel BL/BLI, the available evidence is generally not encouraging. Ray et al. systematically determined the MIC distributions of several new agents against 109 clinical isolates (Ray et al., 2025). For *A. xylosoxidans*, imipenem/relebactam had MIC 50 and MIC 90 values of 2 μg/mL and 4 μg/mL, respectively. Compared with imipenem alone, the addition of relebactam did not substantially lower the MIC values. Ceftazidime/avibactam, meropenem/vaborbactam, and cefiderocol also showed limited activity against this organism (Isler et al., 2020).

Novel tetracycline derivatives showed differential activity. Ray et al. evaluated these agents against a collection of 109 clinical isolates representing multiple Achromobacter species, predominantly *A. xylosoxidans* (*n* = 96). They found that delafloxacin (a novel fluoroquinolone) and omadacycline (a novel tetracycline) had limited or no activity against *Achromobacter*, whereas eravacycline showed low MIC values (MIC50/90, 0.5/1 μg/mL), suggesting its potential for treating *Achromobacter* infections. Plazomicin (a novel aminoglycoside) also did not show better activity than traditional aminoglycosides, with MIC50/90 values >4/>4 μg/mL (Ray et al., 2025).

Taken together, these data indicate that the overall incremental benefit of new antimicrobial agents against *Achromobacter* is limited, and the existing agent’s piperacillin-tazobactam, carbapenems, and TMP-SMX remain the cornerstones of empirical therapy (Bayless et al., 2025). However, this statement requires stratification. In non-CF patients with bloodstream infection or pneumonia, these agents represent reasonable first-line choices when the isolate is susceptible (Bayless et al., 2025). In CF patients with chronic airway infection, the evidence is weaker, because standard susceptibility testing of planktonic cells may not reflect the tolerance of biofilm-embedded populations, and clinical outcome data from randomized trials are lacking (Isler et al., 2020; Abbott and Peleg, 2015). For non-xylosoxidans species, susceptibility profiles are generally more favorable, but species-specific breakpoints are unavailable for most agents, which limits confident clinical interpretation (Ray et al., 2025). Abbott et al. noted that in the absence of clinical outcome data, treatment recommendations should be based on susceptibility reports and pharmacokinetic/pharmacodynamic profiles (Abbott and Peleg, 2015). Isler et al. grouped *Achromobacter* together with *Stenotrophomonas maltophilia* and non-melioidosis *Burkholderia* species as non-fermentative bacteria that pose a high therapeutic challenge. Currently, optimization of novel BL/BLI combinations, efflux pump inhibitors, phage therapy, and other non-classical strategies are still under exploration. More preclinical and clinical studies are needed to verify their efficacy and safety (Isler et al., 2020).

### 4.4 Within-host evolution of resistance

*Achromobacter* can acquire resistance during long-term persistence in the airways of CF patients. A longitudinal analysis of 101 *Achromobacter* genomes from 51 CF patients by Gabrielaite et al. revealed key evolutionary patterns: large deletions in genes encoding negative regulators of efflux pumps, leading to constitutive overexpression of these pumps, and stepwise accumulation of mutations in fluoroquinolone target genes (*parC* and *parE*) (Gabrielaite et al., 2021). Khademi et al. performed whole-genome sequencing and phenotypic analysis of 55 clinical isolates and further confirmed that genetic changes occur mainly in regulatory loci (Khademi et al., 2021). These changes promote the emergence of adaptive phenotypes within the host, such as increased antibiotic resistance and enhanced biofilm formation. This within-host evolution not only drives a gradual rise in resistance levels, but may also co-occur with other adaptive phenotypes, including enhanced biofilm formation and loss of motility. At present, the complete molecular pathways of within-host resistance evolution (from gene mutations to the quantitative resistance phenotype), the similarities and differences in evolutionary trajectories under different antibiotic selective pressures, and potential intervention points in the evolutionary process remain to be systematically clarified (Gabrielaite et al., 2021; Khademi et al., 2021).

### 4.5 Biofilm-mediated tolerance and persister cell formation

The preceding discussion of acquired resistance (Section 4.2) and within-host evolution (Section 4.4) has focused primarily on genetically encoded resistance mechanisms. However, in chronic infections, bacterial pathogens including *Achromobacter* frequently exist not only as planktonic cells but also within biofilm communities and in metabolically heterogeneous states, including persister cells. These features contribute to antimicrobial tolerance and treatment failure through mechanisms that are distinct from conventional, genetically encoded resistance (Isler et al., 2020; Khademi et al., 2021).

Biofilm formation by *Achromobacter* is well documented in clinical isolates from CF patients. The enhancement of biofilm formation during chronic airway infection has been linked to the trade-off with motility loss, as described in Section 5.4, suggesting that this phenotypic transition is part of a broader adaptive program in the host environment (Chalhoub et al., 2022). *Achromobacter* biofilms display increased tolerance to aminoglycosides, fluoroquinolones, and carbapenems compared with planktonic cells, even in the absence of resistance-conferring mutations (Chalhoub et al., 2022). As observed in other biofilm-forming Gram-negative pathogens, this tolerance arises from multiple factors, including limited antibiotic penetration through the biofilm matrix, nutrient and oxygen gradients that generate slow-growing subpopulations, and the presence of persister cells that can survive high concentrations of antibiotics and repopulate the biofilm upon treatment cessation (Isler et al., 2020).

Persister cell formation, a phenomenon well characterized in *Pseudomonas aeruginosa* and other non-fermenting Gram-negative bacteria, has also been demonstrated in clinical *A. xylosoxidans* isolates under conditions of nutrient limitation and antibiotic exposure. In other species, persister formation has been linked to toxin-antitoxin systems, stringent response pathways, and the GacS/GacA two-component regulatory system. The presence of orthologous systems in *Achromobacter* genomes suggests that persister formation may contribute to the recalcitrance of chronic *Achromobacter* infection, although direct experimental evidence in this genus is currently lacking (Khademi et al., 2021).

From a clinical perspective, standard antimicrobial susceptibility testing performed on planktonic cells may substantially underestimate the *in vivo* antibiotic tolerance of biofilm-embedded and persister populations. This discrepancy between *in vitro* susceptibility data and *in vivo* treatment outcomes is well recognized across chronic respiratory infections, including those caused by *P. aeruginosa* and members of the *Burkholderia cepacia* complex, and likely extends to *Achromobacter* (Kerem et al., 2023; Bar-On et al., 2025). Therefore, future studies addressing the metabolic states of bacteria in chronic infection are warranted across multiple chronic infection settings, including CF, and the development of biofilm-specific and persister-targeting therapeutic approaches represents an important direction for future anti-infective research.

## 5 Virulence factors and host adaptation

### 5.1 Secretion systems: molecular basis of pathogenic potential

*Achromobacter* species possess diverse secretion systems, which are an important molecular basis for their role as opportunistic pathogens. A comprehensive analysis of over 200 publicly available genomes by Acharya et al. revealed that *A. xylosoxidans* carries a full spectrum of secretion systems from type I to type VI. Among these is a YscN family type III secretion system (T3SS) that is highly similar to homologous systems in *Bordetella pertussis* and *Pseudomonas aeruginosa*. The T3SS is a key apparatus used by Gram-negative pathogens to inject effector proteins directly into the cytoplasm of host cells, and its presence suggests that *Achromobacter* has the potential to actively manipulate host cell signaling pathways (Acharya et al., 2026).

Pickrum et al. systematically characterized the replication cycle of *A. xylosoxidans* in macrophages and identified associated virulence genes. They found that this bacterium can survive and replicate within macrophages, suggesting that it can evade host innate immune clearance mechanisms. This work provides important evidence for understanding the intracellular pathogenic strategies of *Achromobacter* (Pickrum et al., 2022).

### 5.2 Motility and biofilm formation: dual strategies for environmental adaptation and host colonization

Motility conferred by peritrichous flagella is an important virulence factor of *Achromobacter* and plays a key role in chemotaxis, adhesion, and initial colonization. Studies have confirmed that flagella-mediated motility is one of the key pathogenic phenotypes of this bacterium (Jakobsen et al., 2013).

Filipic et al. compared virulence markers between CF (*n* = 32) and non-CF (*n* = 37) isolates and found that swimming motility was markedly reduced in CF isolates (*p* = 0.044). However, no significant correlation was detected between the swimming phenotype and biofilm formation (correlation coefficient not reported). This phenotypic difference suggests that the selective pressures within the CF airway may favor retention of biofilm formation capacity while selecting against motility (Filipic et al., 2017).

In the chronic infection environment of the CF airway, the flagellar phenotype of *Achromobacter* undergoes notable within-host evolution. A comparative analysis of longitudinal serial isolates from the same patient by Acharya et al. showed that with prolonged infection (over a 2.5-year period, based on three sequential isolates from the same patient), some strains lost or showed a marked reduction in flagellar motility, but gained enhanced biofilm formation capacity and higher levels of resistance through phenotypic trade-offs. Sequence analysis further revealed a tyrosine-to-histidine mutation (Y330H) near the FlhF GTP-binding domain in isolate NIH-016-3 (and subsequently in NIH-016-1), which may affect the flagellar assembly process (Acharya et al., 2026). Sandri et al. analyzed 95 *Achromobacter* isolates from 38 CF patients and reported heterogeneity in biofilm formation and other pathogenic traits between chronic and occasional infection groups, supporting the link between phenotypic diversity and persistent infection (Acharya et al., 2026). Veschetti et al. subsequently used eight of these isolates selected based on these pathogenic characteristics to evaluate *in vivo* inflammatory responses (Sandri et al., 2023).

### 5.3 Induction of inflammatory responses: complexity of the host response

Sahl et al. compared the pro-inflammatory effects of bacterial culture supernatants (exoproducts, containing both secreted and surface-released products) from the four most common *Achromobacter* species in CF (*A. xylosoxidans*, *A. dolens*, *A. insuavis*, and *A. ruhlandii*) on the CF bronchial epithelial cell line CFBE41o and whole blood leukocytes. The results showed that all tested species, including *A. insuavis*, induced significant secretion of IL-6 and IL-8 from CF lung epithelial cells, and the levels of induction were comparable to or even stronger than those induced by *Pseudomonas aeruginosa*. In leukocyte activation experiments, all four *Achromobacter* species activated neutrophils and monocytes in a lipopolysaccharide (LPS)-independent manner (Sahl et al., 2023).

Sandri et al. further demonstrated that eight clinical isolates with different pathogenic characteristics (virulence in Galleria mellonella larvae, cytotoxicity in human bronchial epithelial cells, and biofilm formation) induced varying degrees of inflammatory responses in wild-type and CFTR-knockout [C57BL/6 J Cftr^tm1UNCTgN (FABPCFTR)] mice, and that pathogenic traits such as virulence and cytotoxicity may be associated with clinically relevant outcomes (Sandri et al., 2023). These findings support the role of *Achromobacter* as a CF airway pathogen from an immunopathological perspective.

### 5.4 Genome plasticity and within-host evolution

Core genome analysis of over 200 publicly available genomes revealed that *A. xylosoxidans* has a relatively small core genome, suggesting high genome plasticity and adaptability (Acharya et al., 2026). This plasticity is reflected in the marked phenotypic variation among clinical isolates in cytotoxicity, flagellar motility, biofilm formation capacity, and resistance levels.

Ridderberg et al. performed pioneering work on the genetic adaptation of *Achromobacter* during persistent lung infection in CF patients. They found that many virulence genes needed at the initial stage of acute infection, including type I and type III secretion system genes and genes for pilus and flagellum formation, were under negative selection during chronic infection (Ridderberg et al., 2015). Gabrielaite et al. further revealed genome-wide mutational signatures of within-host adaptive evolution (Gabrielaite et al., 2021). Overall, the main trends in within-host evolution can be summarized as loss or reduction of function in motility-related systems such as flagellar assembly genes, together with increased resistance and enhanced biofilm formation. This trade-off among motility, biofilm formation, and resistance was termed evolutionary trade-off in the study by Khademi et al. and Ridderberg et al. also noted that similar adaptive patterns may extend to other non-fermentative Gram-negative bacilli. Together, these findings suggest that such trade-offs may represent a common pattern of adaptive evolution for pathogens that cause chronic infection in the CF airway (Gabrielaite et al., 2021; Khademi et al., 2021; Ridderberg et al., 2015).

### 5.5 Interspecies interactions: the “dialogue” with *Pseudomonas aeruginosa*

In the polymicrobial airway ecosystem of CF patients, interspecies interactions can have a considerable impact on overall pathogenic outcomes. Besse et al. provided the first *in vivo* evidence for the interaction between *Achromobacter* and *Pseudomonas aeruginosa*. In a zebrafish model, the co-isolated *A. xylosoxidans* strain Ax 200 significantly reduced the virulence of *P. aeruginosa*. Proteomic analysis revealed that Ax 200 altered the expression levels of multiple proteins in *P. aeruginosa* involved in biofilm formation, swimming motility, iron acquisition, and secretion systems (Besse et al., 2025).

Sahl et al. further confirmed bidirectional growth inhibition and biofilm regulation between these two pathogens. These findings reveal the complexity of pathogen-pathogen interactions in the CF airway microbiome from the perspective of interspecies chemical dialogue, and they may offer possible entry points for novel intervention strategies based on the principle of interspecies competition (Sahl et al., 2025).

## 6 Laboratory diagnostic challenges and recommendations

Based on the evidence presented above, the laboratory diagnosis of *Achromobacter* faces several challenges, including species identification, resistance interpretation, and breakpoint availability. We propose a tiered response strategy to address these issues.

### 6.1 We propose a tiered response strategy

*Tier 1*: Optimize the species identification workflow. Clinical microbiology laboratories should use MALDI-TOF MS together with customized expanded databases that cover all known clinically relevant *Achromobacter* species, in order to improve species-level identification accuracy (Papalia et al., 2020). When MALDI-TOF results are uncertain or high-confidence species identification is required, *nrdA* gene sequencing should be used for confirmation. Ray et al. clearly recommended that, given the insufficient spectral coverage in commercial MALDI-TOF system databases, molecular confirmation methods should be selectively adopted in key scenarios that involve treatment decisions (Ray et al., 2025).

*Tier 2*: Promote the establishment of clinical breakpoints. Although EUCAST has established species-specific breakpoints for *A. xylosoxidans* for piperacillin-tazobactam, meropenem, and trimethoprim-sulfamethoxazole, breakpoints for other *Achromobacter* species and for additional antimicrobial classes remain unavailable (Isler et al., 2020; Ray et al., 2025). The currently used “other non-Enterobacterales” breakpoint criteria may not accurately reflect the *in vivo* pharmacodynamic characteristics of non-xylosoxidans species. Establishing additional species-specific MIC clinical breakpoints based on large multicenter clinical data is a key step in linking *in vitro* susceptibility data to individualized clinical decisions. The distinction between EUCAST clinical breakpoints, ECOFFs, tentative ECOFFs (TECOFFs), and CLSI “other non-Enterobacterales” criteria should be clearly communicated in laboratory reports (Table 1).

**Table 1:** Comparison of current breakpoint criteria for *Achromobacter* species.

Criterion	Source	Scope	Details
CLSI “other non-Enterobacterales”	CLSI M100 (Ray et al., 2025)	All *Achromobacter* species	Applied when no species-specific breakpoints exist; may not reflect true pharmacodynamics for *Achromobacter*
EUCAST species-specific breakpoints	EUCAST Breakpoint Tables v16.1 (Isler et al., 2020)	*A. xylosoxidans* only	Piperacillin-tazobactam, meropenem, trimethoprim-sulfamethoxazole
EUCAST PK/PD breakpoints	EUCAST Breakpoint Tables (Isler et al., 2020)	All *Achromobacter* species	Used for agents without clinical breakpoints (e.g., cefiderocol)
Tentative ECOFFs (TECOFFs)	EUCAST (Isler et al., 2020)	*A. xylosoxidans*	Epidemiological cutoff values to distinguish wild-type from non-wild-type populations

*Tier 3*: Implement routine WGS surveillance. WGS can provide a “one-stop” solution for species identification, resistance gene screening, and molecular epidemiological tracing. It is of particular value for investigating multidrug-resistant strains or suspected outbreaks (Ray et al., 2025). Wider application of this technology platform at the reference laboratory level will strengthen clinical response capacity.

## 7 Emerging therapeutic strategies

### 7.1 Phage therapy: from proof of concept to clinical application

Given the intrinsic and acquired resistance of *Achromobacter* to most antimicrobial agents, phage therapy is seen as a highly promising alternative treatment strategy. Cobián Güemes et al. built the most systematic framework for *Achromobacter* phage therapy to date. They identified 13 lytic phages and assembled the Kumeyaay phage collection, which covers 6 distinct genomic clusters. This collection showed lytic activity against 78% of 23 tested clinical isolates. The researchers used octanol extraction to prepare a phage concentrate with an endotoxin concentration of 65 EU/mL at a titer of 1 × 10^9^ PFU/mL. Whether this preparation meets regulatory endotoxin limits depends on the intended dose, route of administration, infusion period, and patient body weight, because FDA endotoxin limits are calculated as 5 EU/kg for non-intrathecal administration and are therefore dose-dependent rather than based on a single concentration threshold (Cobian Guemes et al., 2023). This work provides a preliminary framework for producing clinical-grade phage preparations, but additional quality control standards are needed.

Phage therapy has shown promise in preclinical models and in one reported clinical case. Lebeaux et al. reported the first case of phage treatment for a pandrug-resistant *A. xylosoxidans* infection in a CF child after lung transplantation. Their study revealed that the dynamics of airway colonization are far more complex than bacterial culture results suggest, involving the coexistence of phage-susceptible and phage-resistant strains (Lebeaux et al., 2021). However, this is a single case report, and several obstacles must be addressed before phage therapy can be considered for wider clinical use. Cobián Güemes et al. observed that during preparation of lytic phages, the *A. xylosoxidans* strain CF418 could be induced to produce the lysogenic phage CF418-P1 (Cobian Guemes et al., 2023). This suggests that lysogenic phages may mediate horizontal transfer of virulence and resistance genes. Such a finding imposes strict requirements on the quality control of phage preparations. In addition, the narrow host range of phages is both an advantage for precision therapy and a limitation, as it usually requires customized phage cocktails. This poses operational challenges for large-scale production and rapid matching. Furthermore, phage resistance can emerge rapidly during treatment, the pharmacokinetics of phages in human airways remain poorly characterized, and regulatory frameworks for phage therapy vary widely across jurisdictions. These factors complicate the path from compassionate use to randomized controlled trials (Cobian Guemes et al., 2023; Lebeaux et al., 2021).

### 7.2 New antimicrobial agents and efflux pump inhibitors

In the field of conventional antimicrobial agents, recent data suggest that resources should not be overly concentrated on extending the indications of existing β-lactam/β-lactamase inhibitor combinations. Data from Ray et al. showed that relebactam had unsatisfactory inhibitory activity against *A. xylosoxidans* β-lactamases. Eravacycline has emerged as a candidate worth watching because of its low MIC values (Ray et al., 2025). However, its clinical efficacy and safety in patients with *Achromobacter* infections have not been systematically evaluated, and more clinical data are needed (Ray et al., 2025). A more promising direction may be the development of efflux pump inhibitors. Given the central role of RND-type efflux pumps such as AxyXY-OprZ in the intrinsic resistance of *Achromobacter*, Chalhoub et al. have already provided an experimental basis for targeting efflux pumps (Chalhoub et al., 2022). If clinically usable efflux pump inhibitors can be found and used in combination, they could theoretically restore the activity of many existing antimicrobial agents against *Achromobacter*.

## 8 Discussion

This review synthesizes current evidence on the taxonomy, identification, clinical epidemiology, resistance mechanisms, virulence factors, diagnostic strategies, and emerging therapies for *Achromobacter*. Several themes deserve critical discussion.

First, the evidence base is uneven. Most mechanistic studies and clinical data focus on *A. xylosoxidans* in CF populations. Non-xylosoxidans species and non-CF infections remain understudied, which limits the generalizability of current recommendations. Future research should expand species-level surveillance in non-CF immunocompromised hosts and in non-xylosoxidans species.

Second, the causal link between *Achromobacter* detection and clinical decline in CF is supported by longitudinal association studies, but confounding by disease severity, co-infection, and treatment exposure remains a concern. The dose–response pattern observed by Bar-On et al. strengthens the argument for pathogenicity (Bar-On et al., 2025), yet randomized intervention trials are lacking. Without them, the question of whether aggressive eradication improves long-term outcomes remains unanswered.

Third, the ‘motility-biofilm-resistance’ framework presented in this review builds on prior work by Khademi et al. and Acharya et al., who identified individual components of these trade-offs (Acharya et al., 2026; Khademi et al., 2021). Here we synthesize these observations into a unified model that links phenotypic shifts to clinical tolerance (Figure 1 provides a schematic summary of this integrated framework). We acknowledge that not all longitudinal isolates follow the same trajectory; some retain motility, and biofilm formation varies by strain and host environment (Figure 1 indicates this alternative trajectory with a dashed box). The framework should therefore be viewed as a pattern rather than a universal rule.

**Figure 1:**
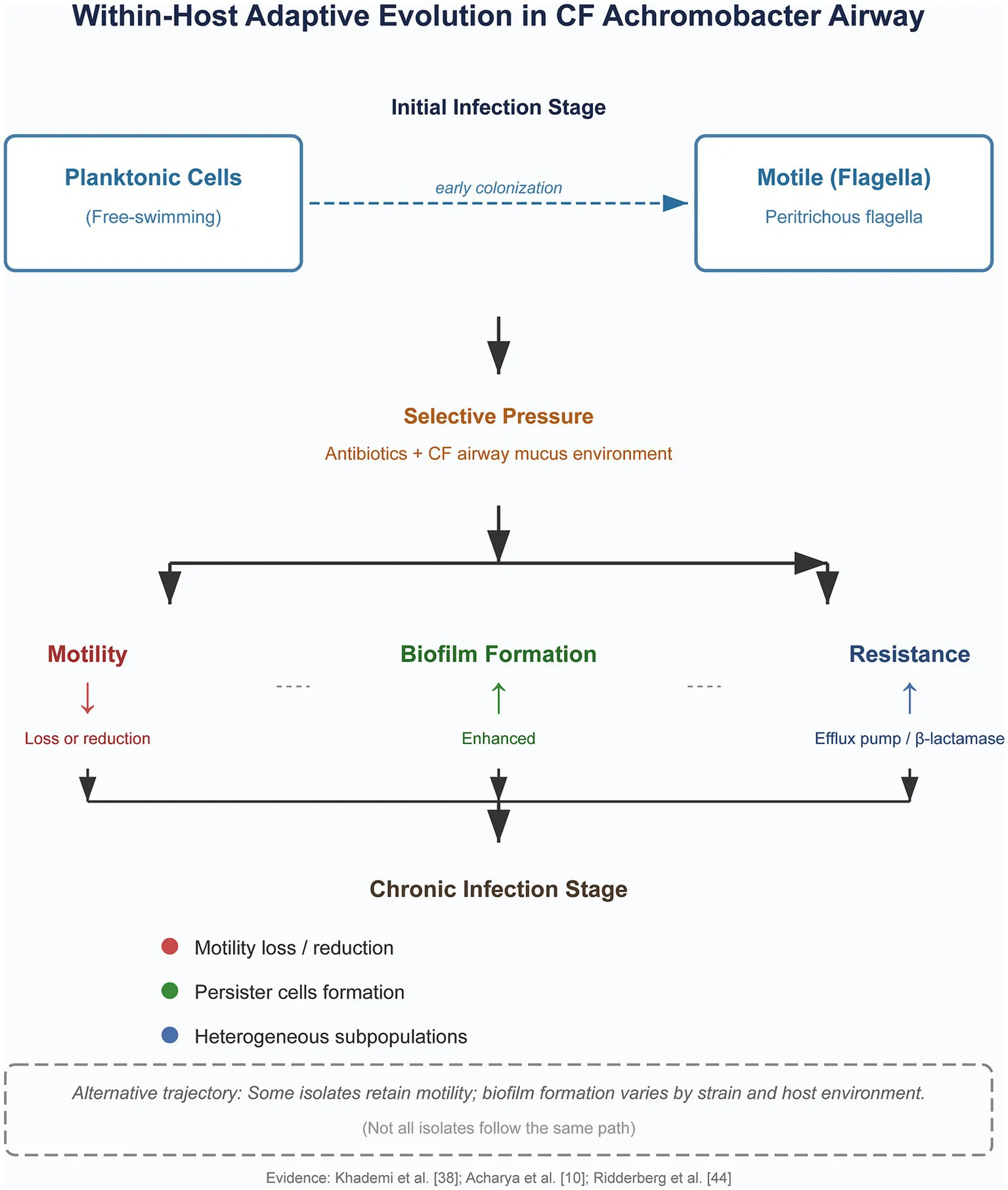
Schematic illustration of the within-host adaptive evolution of *Achromobacter* in the cystic fibrosis airway. The model depicts the transition from initial infection (planktonic, motile cells) to chronic infection, driven by selective pressures (antibiotics and CF airway environment). Key phenotypic shifts include reduced motility (↓), enhanced biofilm formation (↑), and increased resistance (↑). The dashed box indicates that not all longitudinal isolates follow the same trajectory. Evidence from Khademi et al. (2021), Acharya et al. (2026), and Ridderberg et al. (2015) supports the individual components of this framework.

Fourth, diagnostic advances are outpacing clinical breakpoints. MALDI-TOF MS databases and WGS can now identify most species, but EUCAST breakpoints exist only for *A. xylosoxidans* and for a limited number of agents. This gap creates interpretive uncertainty for non-xylosoxidans species and for newer agents such as cefiderocol.

Finally, therapeutic options remain limited. Novel BL/BLI combinations show modest activity, and phage therapy remains experimental. Efflux pump inhibitors represent a rational target, but no compound has reached clinical trials for *Achromobacter*. Until these tools mature, optimization of existing agents through species-level identification, pharmacokinetic monitoring, and biofilm-targeting strategies remains the most practical approach.

## 9 Future perspectives and conclusions

The genus *Achromobacter* is undergoing a shift in status from a rare pathogen to a clinically important opportunistic pathogen. This review has systematically examined research progress in taxonomy, clinical epidemiology, resistance mechanisms, virulence factors, diagnostic strategies, and emerging therapies. This work reveals a multi-layered picture. Technical challenges in accurate species identification, synergistic barriers posed by multiple resistance mechanisms, the dynamic nature of within-host adaptive evolution, and the ecological complexity of interspecies interactions together contribute to current difficulties in clinical management and define the frontiers of future research.

Based on the analysis presented here, priority research directions can be summarized as follows.

First, efforts to update MALDI-TOF MS databases and standardize species identification workflows are likely to yield substantial clinical benefit. This would mean expanding mass spectral profile coverage to include all known clinically relevant *Achromobacter* species and integrating molecular confirmation methods into standardized diagnostic protocols (Papalia et al., 2020; Garrigos et al., 2022).

Second, the establishment of species-specific clinical breakpoints remains an important goal, but will require international multicenter collaboration that brings together MIC distribution data, pharmacokinetic/pharmacodynamic models, and clinical outcome data, so as to replace the currently imprecise “other non-Enterobacterales” breakpoint reference (Isler et al., 2020; Ray et al., 2025). The recent establishment of tentative ECOFFs by EUCAST for *A. xylosoxidans* represents a first step, but breakpoints for other species and agents are still needed.

Third, further investigation into the regulatory networks governing resistance and virulence is warranted. Multi-omics approaches can be used to systematically dissect the regulatory mechanisms controlling efflux pump and β-lactamase expression, as well as the molecular switches that underlie the motility-biofilm-resistance phenotypic trade-off. Such work may eventually provide a theoretical basis for identifying new drug targets (Acharya et al., 2026; Chalhoub et al., 2022; Khademi et al., 2021).

Fourth, the clinical development of phage therapy could be advanced in a standardized manner, moving from single case reports toward randomized controlled clinical trials. Quality control standards for phage preparations need to be established, and the synergistic effects of phage-antibiotic combination regimens should be systematically evaluated (Cobian Guemes et al., 2023; Lebeaux et al., 2021). However, the path to regulatory approval and widespread clinical implementation remains uncertain.

Fifth, interspecies interactions and their clinical consequences merit further exploration. Within the framework of polymicrobial infection in the CF airway, the interaction networks between *Achromobacter* and other pathogens such as *Pseudomonas aeruginosa* may reveal opportunities for novel therapeutic interventions (Besse et al., 2025). However, the clinical relevance of *in vitro* and *in vivo* competition data to patient outcomes remains to be demonstrated.

Research on *Achromobacter* is at a critical juncture. Technical challenges are gradually being resolved, clinical evidence is building, and therapeutic approaches are showing promise. Interdisciplinary and international collaboration will be essential to move this field from descriptive knowledge toward mechanistic understanding and precision medicine.
